# Early Viral Clearance and Antibody Kinetics of COVID-19 Among Asymptomatic Carriers

**DOI:** 10.3389/fmed.2021.595773

**Published:** 2021-03-15

**Authors:** Tongyang Xiao, Yanrong Wang, Jing Yuan, Haocheng Ye, Lanlan Wei, Xuejiao Liao, Haiyan Wang, Shen Qian, Zhaoqin Wang, Lei Liu, Zheng Zhang

**Affiliations:** ^1^National Clinical Research Center for Infectious Disease, School of Medicine, Institute of Hepatology, Shenzhen Third People's Hospital, The Second Affiliated Hospital, Southern University of Science and Technology, Shenzhen, China; ^2^Department of Pediatrics, National Clinical Research Center for Infectious Disease, Shenzhen Third People's Hospital, Shenzhen, China; ^3^Department of Infectious Diseases, National Clinical Research Center for Infectious Disease, Shenzhen Third People's Hospital, Shenzhen, China; ^4^Shenzhen Research Center for Communicable Disease Diagnosis and Treatment of Chinese Academy of Medical Science, Shenzhen, China; ^5^Guangdong Key Laboratory for Anti-infection Drug Quality Evaluation, Shenzhen, China

**Keywords:** asymptomatic, viral clearance, antibody, SARS-CoV-2, COVID-19

## Abstract

Asymptomatic carriers contribute to the spread of Coronavirus Disease 2019 (COVID-19), but their clinical characteristics, viral kinetics, and antibody responses remain unclear. A total of 56 COVID-19 patients without symptoms at admission and 19 age-matched symptomatic patients were enrolled. RNA of SARS-CoV-2 was tested using transcriptase quantitative PCR, and the total antibodies (Ab), IgG, IgA, and IgM against the SARS-CoV-2 were tested using Chemiluminescence Microparticle Immuno Assay. Among 56 patients without symptoms at admission, 33 cases displayed symptoms and 23 remained asymptomatic throughout the follow-up period. 43.8% of the asymptomatic carriers were children and none of the asymptomatic cases had recognizable changes in C-reactive protein or interleukin-6, except one 64-year-old patient. The initial threshold cycle value of nasopharyngeal SARS-CoV-2 in asymptomatic carriers was similar to that in pre-symptomatic and symptomatic patients, but the positive viral nucleic acid detection period of asymptomatic carriers (9.63 days) was shorter than pre-symptomatic patients (13.6 days). There were no obvious differences in the seropositive conversion rate of total Ab, IgG, and IgA among the three groups, though the rates of IgM varied largely. The average peak IgG and IgM COI of asymptomatic cases was 3.5 and 0.8, respectively, which is also lower than those in symptomatic patients with peaked IgG and IgM COI of 4.5 and 2.4 (*p* < 0.05). Young COVID-19 patients seem to be asymptomatic cases with early clearance of SARS-CoV-2 and low levels of IgM generation but high total Ab, IgG, and IgA. Our findings provide empirical information for viral clearance and antibody kinetics of asymptomatic COVID-19 patients.

## Introduction

An outbreak of the 2019 novel coronavirus disease (COVID-19) has garnered international attention, rapidly spreading across the globe since it was first diagnosed in December of 2019. The World Health Organization (WHO) has confirmed more than 112 million COVID-19 cases worldwide, resulting in approximately 2.49 million deaths, as of February 25, 2021 (1). The infectiousness and transmission of the COVID-19 are particularly worrisome to public health officials, as some cases have spread asymptomatically. Asymptomatic transmission of severe acute respiratory syndrome coronavirus 2 (SARS-CoV-2) has been documented (2, 3), with the proportion of cases attributed to asymptomatic transmission varying widely, with rates ranging from 1.2 to 50% (4, 5). Asymptomatic infection refers to a person who has no clinical symptoms (such as fever, cough, or sore throat), yet test positive for the virus or serum antibody against SARS-CoV-2 (6). SARS-CoV-2 has the possibility of viral shedding, thus transmission of the virus can occur during the asymptomatic period (7–9). Patients can be infected and transmit the disease without showing symptoms, suggesting that perhaps further isolation and continuous nucleic acid testing may be warranted after a patient is discharged (3). As no vaccine has yet to be developed and treatment options are limited, identifying and containing the spread of these asymptomatic infections are key interventions that are necessary if governments and healthcare systems are to control the spread of COVID-19 and reduce disease-related mortality.

Because asymptomatic patients are difficult to track and find, they may contribute to the spread of infection and even lead to secondary outbreaks. Therefore, the screening and diagnosing of asymptomatic carriers, particularly in the early phase of infection, are beneficial for the prevention and treatment of COVID-19. Since the virus are always waiting for opportunities to reproduce and invade organisms, the asymptomatic infections are possibly be flared up when immune responses were weakened. Thus, this study aimed to understand its mechanism, viral clearance, and antibody kinetics of asymptomatic carriers to better inform national control policies and prevent further infection.

## Results

### Demographic Characteristics of Asymptomatic SARS-CoV-2 Infected Patients

**Figure 1:**
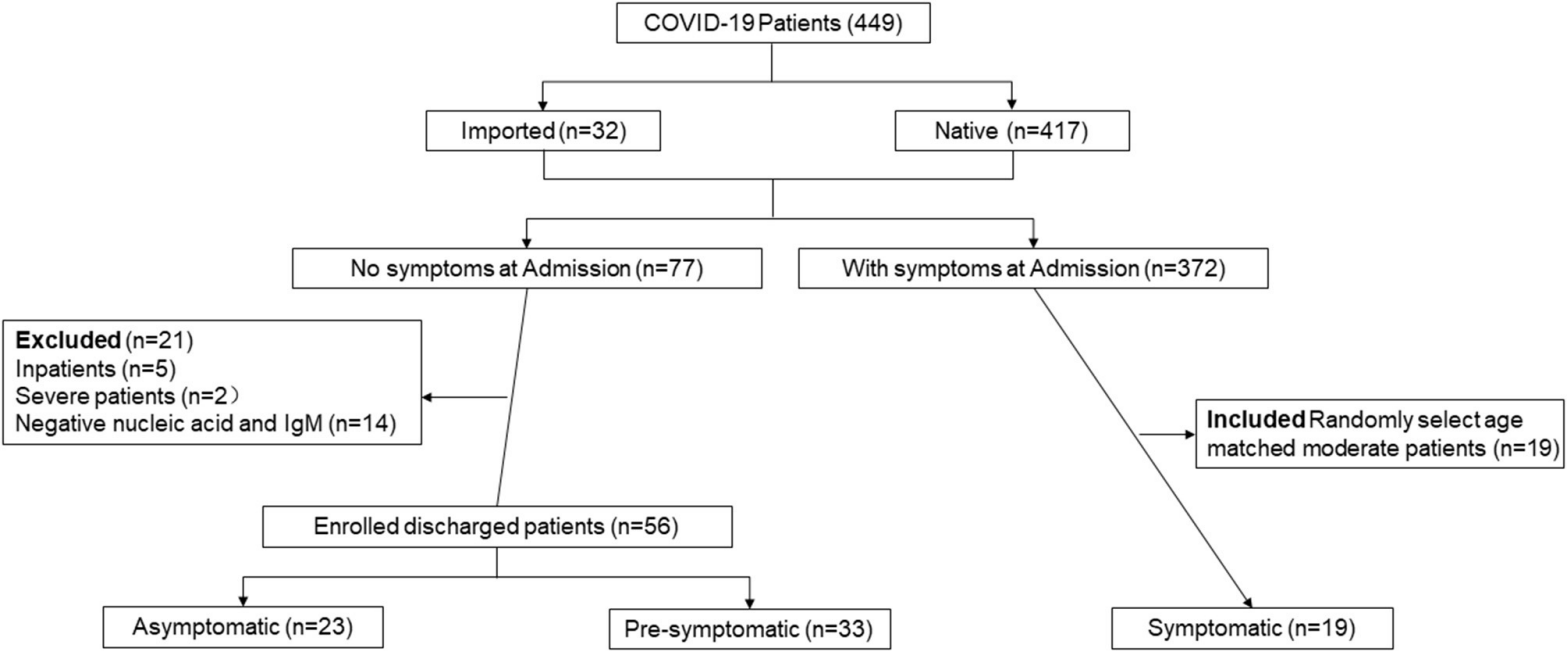
Participant profile. The figure depicts the study design and general inclusion criteria of the study.

Figure 1 summarizes the study design. From January 11 to April 1, 2020, a total of 449 patients (417 native patients and 32 imported cases) were admitted to the Third People's Hospital of Shenzhen. A total of 77 cases (17.15%) without symptoms at admission were enrolled including intimate contacts found by active surveillance and international travelers, but 21 cases were excluded due to severely progressive conditions (*n* = 2), inpatients (*n* = 5), or undetectable RNA and IgM (*n* = 14). Finally, 56 cases without symptoms at admission including 23 asymptomatic and 33 pre-symptomatic were discharged, and were enrolled in the final analysis. Of the 372 cases with symptoms at admission, only 19 moderate patients were aged-matched and were selected as controls.

### Clinical Characteristics of Asymptomatic Patients

As seen in Table 1, asymptomatic carriers were younger compared to pre-symptomatic and symptomatic patients. 43.5% of asymptomatic carriers were children, of whom two were infants, 78.3% of asymptomatic carriers were female, and two (8.7%) asymptomatic carriers had comorbidities, such as hypertension and asthma. 34.8% of asymptomatic carriers and 30.3% of pre-symptomatic patients tested positive for SARS-CoV-2 RNA after discharge. 56.5% of asymptomatic carriers showed abnormal manifestations, while more than 80% of pre-symptomatic patients had abnormal chest CT images, such as ground-glass opacity and bilateral patchy shadowing (*p* < 0.05). Compared with symptomatic patients, the length of hospital stay for asymptomatic and pre-symptomatic patients was slightly longer, but was not found to be significant (*p* = 0.07). All participants received antiviral drugs such as Ritonavir, Chloroquine, Ribavirin, or interferon. And there was little difference in drug options among different groups. And 82.6% of asymptomatic cases and 94.7% of symptomatic patients received interferon, while only 54.6% of pre-symptomatic patients were treated with interferon (*p* = 0.003).

**Table 1:** Clinical information of the enrolled patients.

	**Asymptomatic**	**Presymptomatic**	**Symptomatic**	***p* (*p*1/*p*2/*p*3)**
Number	23	33	19	
Age, median (IQR)	30 (41.8)	45 (30.5)	25 (36.0)	0.19
≤ 14 years old, *n* (%)	10 (43.5)	7 (21.2)	5 (26.3)	
>14 years old, *n* (%)	13 (56.5)	26 (78.8)	14 (73.7)	
Gender, *n* (%)				<0.05 (0.01/0.08/0.62)
Male	5 (21.7)	18 (54.6)	9 (47.4)	
Female	18 (78.3)	15 (45.5)	10 (52.6)	
Comorbidities, *n* (%)	2 (8.7)	9 (27.3)	4 (21.1)	0.23
RP occurrence, *n* (%)	8 (34.8)	10 (30.3)	10 (52.6)	0.26
Abnormal Chest CT imaging, *n* (%)	13 (56.5)	29 (87.9)	16 (84.2)	0.02 (0.0007/0.05/0.71)
Treatment, *n* (%)
Antiviral drugs	20 (87.0)	32 (97.0)	16 (84.2)	0.24
ritonavir	16 (69.6)	24 (72.7)	13 (68.4)	0.12
chloroquine	2 (8.7)	2 (6.1)	1 (5.3)	0.15
ribavirin	2 (8.7)	3 (9.1)	0 (0)	0.08
Interferon therapy	19 (82.6)	18 (54.6)	18 (94.7)	0.003 (0.03/0.23/0.003)
Symptoms, *n* (%)
Fever	0	11 (33.3)	13 (68.4)	0.01
Cough	0	22 (66.7)	13 (68.4)	0.9
Chest tightness	0	2 (6.1)	1 (5.3)	0.91
Ct value at admission (mean ± SD)	29.9 ± 4.8 (*n* = 19)	29.1 ± 6.8 (*n* = 30)	29.2 ± 5.7 (*n* = 15)	0.89
Days of exposure to first positive RNA test (mean ± SD)	16.8 + 6.7 (*n* = 19)	16.6 + 7.9 (*n* = 30)	14.1 + 5.3 (*n* = 15)	0.35
Days of exposure to hospital admission (mean ± SD)	15.0 ± 7.0 (*n* = 21)	14.7 + 7.9 (*n* = 30)	12.5 ± 6.0 (*n* = 19)	0.38
Days from onset to admission (mean ± SD)	1.1 ± 1.2 (*n* = 23)	2.3 ± 3.0 (*n* = 33)	4.1 ± 1.7 (*n* = 19)	0.0005 (0.15/0.0003/0.02)
Days from onset to first positive RNA test (mean ± SD)	3.4+3.7 (*n* = 19)	4.2 ± 3.4 (*n* = 30)	5.9 ± 2.4 (*n* = 15)	0.09
Days of positive viral nucleic acid detection (mean ± SD)^#^	9.6 ± 5.3 (*n* = 19)	13.6 ± 6.6 (*n* = 30)	9.7 ± 4.3 (*n* = 14)	0.03 (0.03/0.96/0.05)
Days from onset to RNA negative-conversion (mean ± SD)^*^	12.1 ± 5.8 (*n* = 19)	16.6 ± 7.5 (*n* = 30)	16.6 ± 5.6 (*n* = 14)	0.05
Days of antiviral treatment (mean ± SD)	12.0 ± 2.8 (*n* = 23)	14.4 ± 5.8 (*n* = 30)	14.4 ± 5.2 (*n* = 19)	0.17
Days of Hospital stays (mean ± SD)	20.3 ± 9.7 (*n* = 23)	23.4 ± 6.9 (*n* = 30)	18.5 ± 5.6 (*n* = 19)	0.07

*Data are n (%), median (interquartile range, IQR) or mean ± SD. RP (Recovered COVID-19 with re-detectable SARS-CoV-2 after discharge), Ct (Threshold cycle)*.

* *Days of RNA negative-conversion is the time since the onset of illness to last RNA negative-conversion, the onset of asymptomatic patients is the time of first actual admission*;

# *positive viral nucleic acid detection is from the first day of positive nucleic acid test to the first day of continuous negative test during hospitalization; Chi-square or Fisher's exact tests were utilized to compare the proportions of the categorical variables. One-way ANOVA or Wilcoxon rank-sum tests were used for the continuous variables. p1, p2, and p3 were comparison between asymptomatic and presymptomatic, asymptomatic and symptomatic, presymptomatic and symptomatic, respectively. p-value < 0.05 indicates significant differences*.

### Laboratory Characteristics of Asymptomatic Patients

Only a few asymptomatic cases had recognizable changes in laboratory tests (Table 2). C-reactive protein (CRP) and interleukin (IL)-6 are two inflammatory markers. At admission, all asymptomatic patients showed normal CRP and IL-6 levels which did not progress, except in one 64-year-old patient whose CRP and IL-6 were slightly increased until discharge. However, elevated CRP occurred in 8 pre-symptomatic and 6 symptomatic participants (*p* < 0.05) and increased IL-6 occurred in 9 pre-symptomatic and 8 symptomatic participants, despite both obtaining normal CRP and IL-6 after treatment (*p* < 0.05). Lactate dehydrogenase (LDH) was used as an indicator of disease severity. During the course of disease, LDH was increased in all of the groups and no significant difference was present in other laboratory tests, such as myohemoglobin (MYO), oxyhemoglobin saturation (O_2_AT) between the three groups. In addition, the average white blood cell (WBC), lymphocyte, CD4+ T cell, and CD8+ T cell counts were slightly changed within normal ranges during the hospitalization period, but no significant difference was observed among the three groups (Supplementary Figure 1).

**Table 2:** Laboratory characteristics of asymptomatic patients.

		**Asymptomatic**	**Presymptomatic**	**Symptomatic**	***p* (*p*1/*p*2/*p*3)**
Elevated PCT, *n* (%)	Before	0/22 (0.0)	0/33 (0.0)	1/18 (5.6)	0.3
≥0.5 ng/mL	Middle	0/22 (0.0)	0/32 (0.0)	1/18 (5.6)	0.3
	After	0/21 (0.0)	0/25 (0.0)	0/16 (0.0)	NA
Elevated CRP, *n* (%)	Before	0/23 (0.0)	8/32 (25.0)	6/18 (33.3)	0.01 (0.02/0.004/0.53)
≥8 mg/L	Middle	0/23 (0.0)	4/33 (12.1)	7/18 (38.9)	0.001 (0.14/0.001/0.04)
	After	1/22 (4.6)	6/26 (23.1)	2/15 (13.3)	0.46
Elevated IL-6, *n* (%)	Before	0/22 (0.0)	9/28 (32.1)	8/16 (50.0)	0.001 (0.003/0.000/0.24)
≥7 pg/mL	Middle	0/21 (0.0)	4/22 (18.2)	3/12 (25.0)	0.05
	After	0/14 (0.0)	2/21 (9.5)	2/11 (18.2)	0.25
Elevated ESR, *n* (%)	Before	6/21 (28.6)	12/32 (37.5)	6/16 (37.5)	0.77
≥20 mm/h	Middle	6/9 (66.7)	5/13 (38.5)	5/5 (100)	0.05
	After	5/13 (38.5)	5/12 (41.7)	5/6 (83.3)	0.19
Elevated LDH, *n* (%)	Before	8/22 (36.4)	11/33 (66.7)	7/18 (38.9)	0.92
≥250 U/L	Middle	3/19 (15.8)	3/28 (10.7)	4/15 (26.7)	0.41
	After	3/18 (16.7)	1/23 (4.4)	1/16 (6.3)	0.5
Elevated MYO, *n* (%)	Before	0/16 (0.0)	0/29 (0.0)	0/13 (0.0)	NA
≥110 ng/mL	Middle	0/14 (0.0)	0/18 (0.0)	0/9 (0.0)	NA
	After	0/15 (0.0)	0/15 (0.0)	0/9 (0.0)	NA
Elevated CK, *n* (%)	Before	0/16 (0.0)	2/27 (7.4)	1/10 (10.0)	0.58
≥200 U/L	Middle	3/14 (21.4)	2/17 (11.8)	0/7 (0.0)	0.57
	After	3/16 (18.8)	2/15 (13.3)	0/6 (0.0)	0.83
Elevated D-DIC, *n* (%)	Before	3/23 (13.0)	3/33 (9.1)	4/18 (22.2)	0.42
≥0.5 μg/mL	Middle	0/11 (0.0)	6/24 (25.0)	5/16 (31.3)	0.11
	After	0/18 (0.0)	6/25 (24.0)	3/15 (20.0)	0.06
Decreased PO2, *n* (%)	Before	6/20 (30.0)	10/31 (32.3)	7/18 (38.9)	0.83
≤ 75 mmHg	Middle	6/18 (33.3)	9/31 (29.0)	8/18 (44.4)	0.55
	After	5/14 (35.7)	5/21 (23.8)	6/16 (37.5)	0.62
Decreased O2AT, *n* (%)	Before	0/20 (0.0)	1/31 (3.2)	0/18 (0.0)	1
≤ 91.9%	Middle	1/18 (5.6)	1/31 (3.2)	1/17 (5.9)	1
	After	0/14 (0.0)	3/21 (14.3)	0/16 (0.0)	0.11
Elevated ALT, *n* (%)	Before	1/23 (4.4)	5/33 (15.2)	1/19 (5.3)	0.4
≥45 U/L	Middle	1/21 (4.8)	2/31 (6.5)	0/18 (0.0)	0.79
	After	1/22 (4.6)	4/28 (14.3)	5/19 (26.3)	0.17
Elevated AST, *n* (%)	Before	3/23 (13.0)	1/33 (3.0)	2/19 (10.5)	0.36
≥45 U/L	Middle	0/21 (0.0)	1/31 (3.2)	1/18 (5.6)	0.73
	After	0/22 (0.0)	0/28 (0.0)	2/19 (10.5)	0.07

*Data are n (%), n represents elevated or decreased cases to total cases. p1, p2, and p3 were comparison between asymptomatic and presymptomatic, asymptomatic and symptomatic, presymptomatic, and symptomatic, respectively. Before was the test done at admission to hospital, middle tests were done after treatment, after tests was done before discharge. Chi-square or Fisher's exact tests were utilized to compare the proportions of the categorical variables. p-value < 0.05 indicates significant differences*.

### Rapid Viral Clearance in Asymptomatic Cases

There was no obvious difference in the calculated initial Ct value of nasopharyngeal samples among the three groups (Table 1). The average viral nucleic acid positive detection of asymptomatic cases was 9.63 days, which was significantly shorter than 13.6 days in pre-symptomatic patients (*p* < 0.05). Interestingly, the symptomatic patients also had a short period of positive viral nucleic acid (9.71 days). Similarly, the days of RNA negative-conversion of asymptomatic patients were slightly short than the other two groups. The average conversion days of asymptomatic, pre-symptomatic, and symptomatic were 12.11, 16.63, and 16.64, respectively.

**Figure 2:**
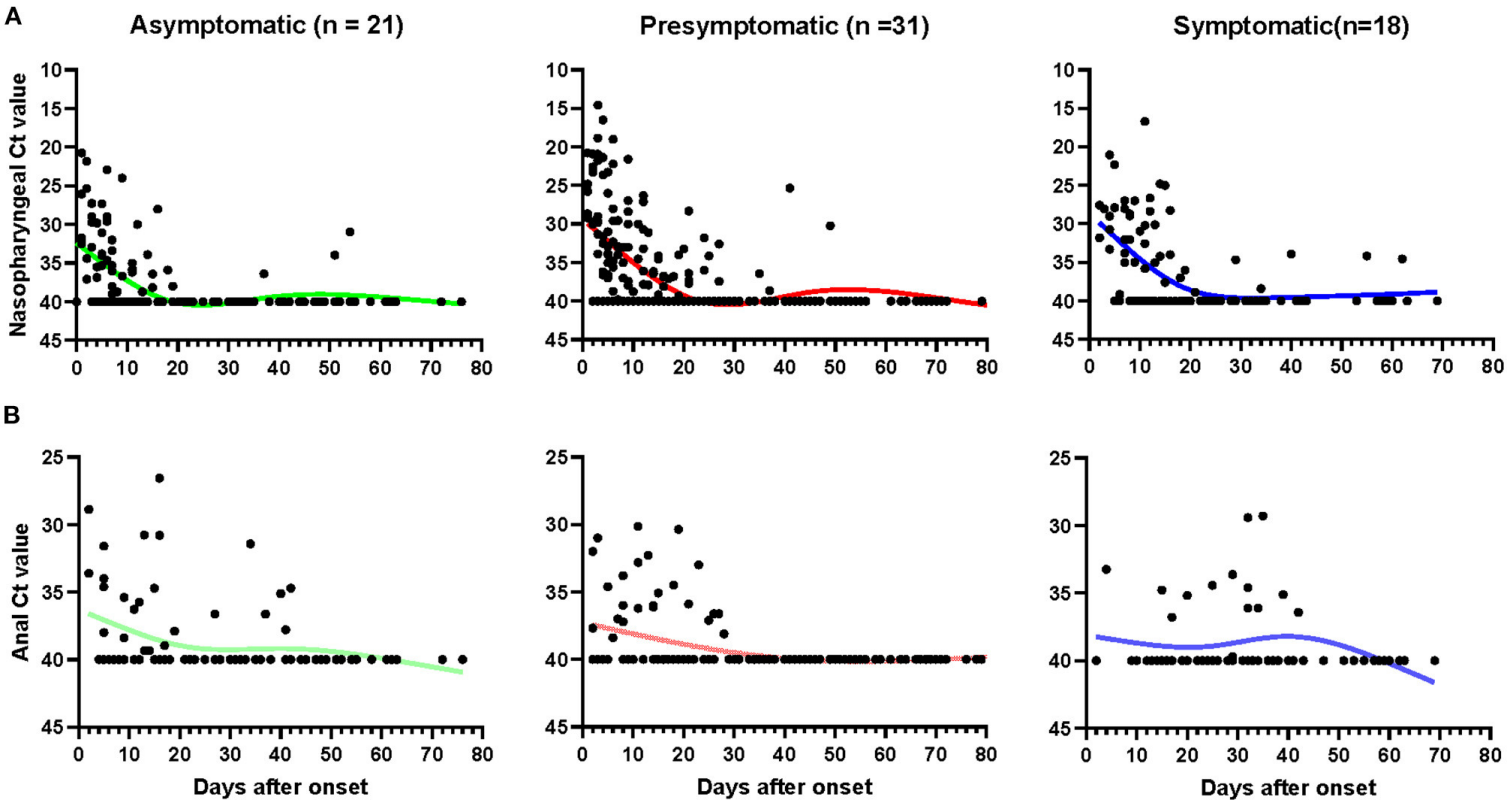
The kinetics of SARS-CoV-2 RNA in nasopharyngeal and anal samples. The threshold cycle (Ct) of nasopharyngeal **(A)** and anal **(B)** samples from patients with positive SARS-CoV-2 RNA test. Patients with totally negative results were not enrolled. Each point represents a sample, curves represent best fit line. Fit Spline/LOWESS of XY analyses with default ones was used for the fitted curve. Negative results are denoted with a Ct of 40.

The kinetics of SARS-CoV-2 RNA in nasopharyngeal and anal samples are shown in Figures 2A,B, respectively. The model-based initial viral load in nasopharyngeal samples of asymptomatic cases was lower, with more than 30 Ct values, as compared to pre-symptomatic and symptomatic cases, which had <30 Ct values. The RNA negative-conversion of asymptomatic cases occurred within 15–20 days after the onset and occurred after 20 days from the onset of admission in pre-symptomatic and symptomatic patients. On the contrary, the initial viral load in anal samples of asymptomatic cases was slightly higher than that in pre-symptomatic and symptomatic patients, and the RNA negative-conversion time was longer than in pre-symptomatic patients. In addition, the re-appearance of SARS-CoV-2 RNA could be observed in nasopharyngeal and anal samples 54 and 42 days, respectively, after the onset of admission for some asymptomatic carriers, 81 and 40 days for pre-symptomatic patients, and 62 and 42 for symptomatic patients, respectively.

### Sero-Conversion of Antibodies Against SARS-CoV-2

**Figure 3:**
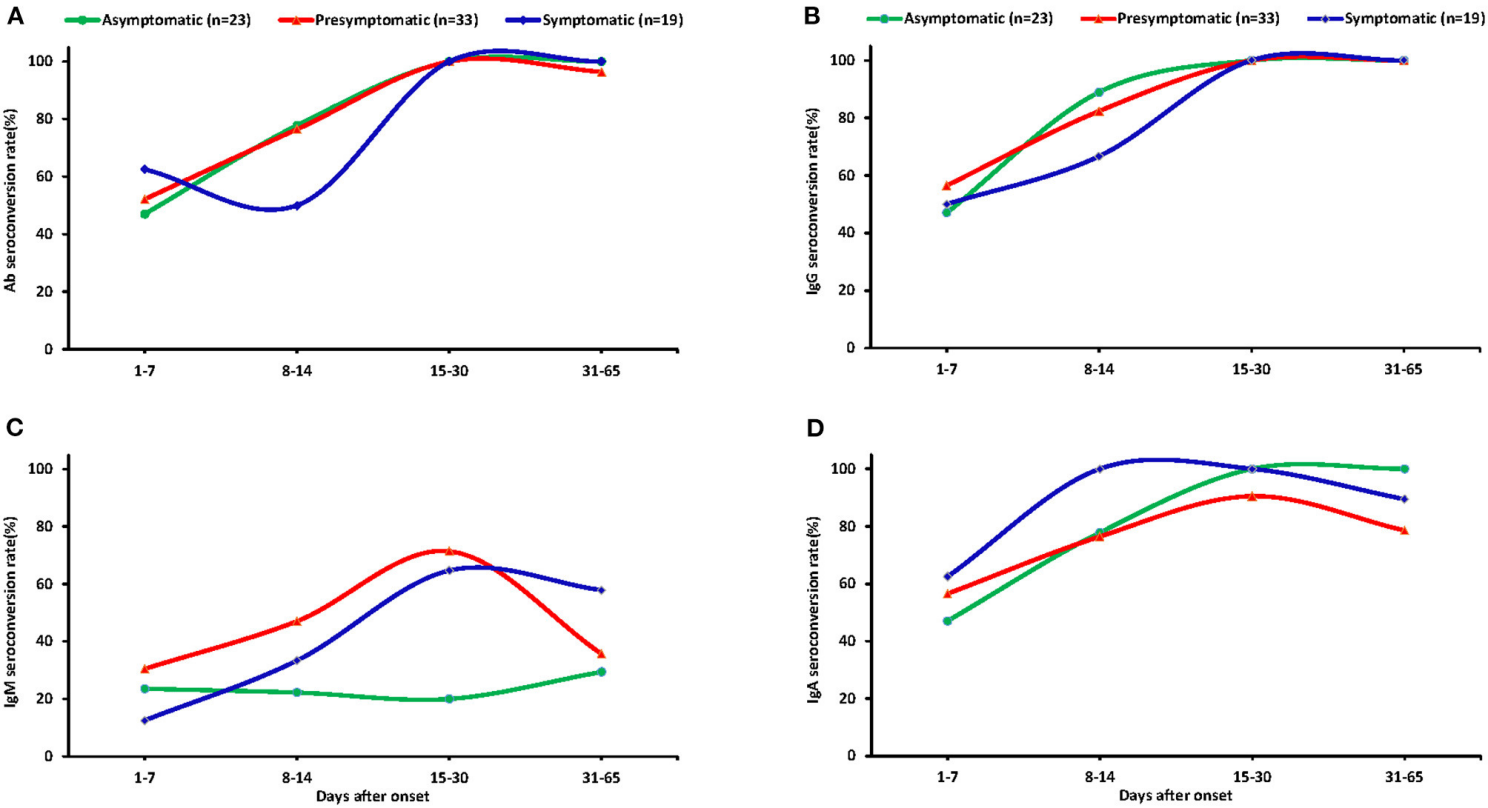
The seroconversion rate of antibodies in the plasma of different patients. Dynamic seropositive rate of total Ab **(A)**, IgG **(B)**, IgM **(C)**, and IgA **(D)** of different patients at different stage. Patients were divided into four groups: 1–7 days, 8–14 days, 15–30 days, and 31–65 days.

**Figure 4:**
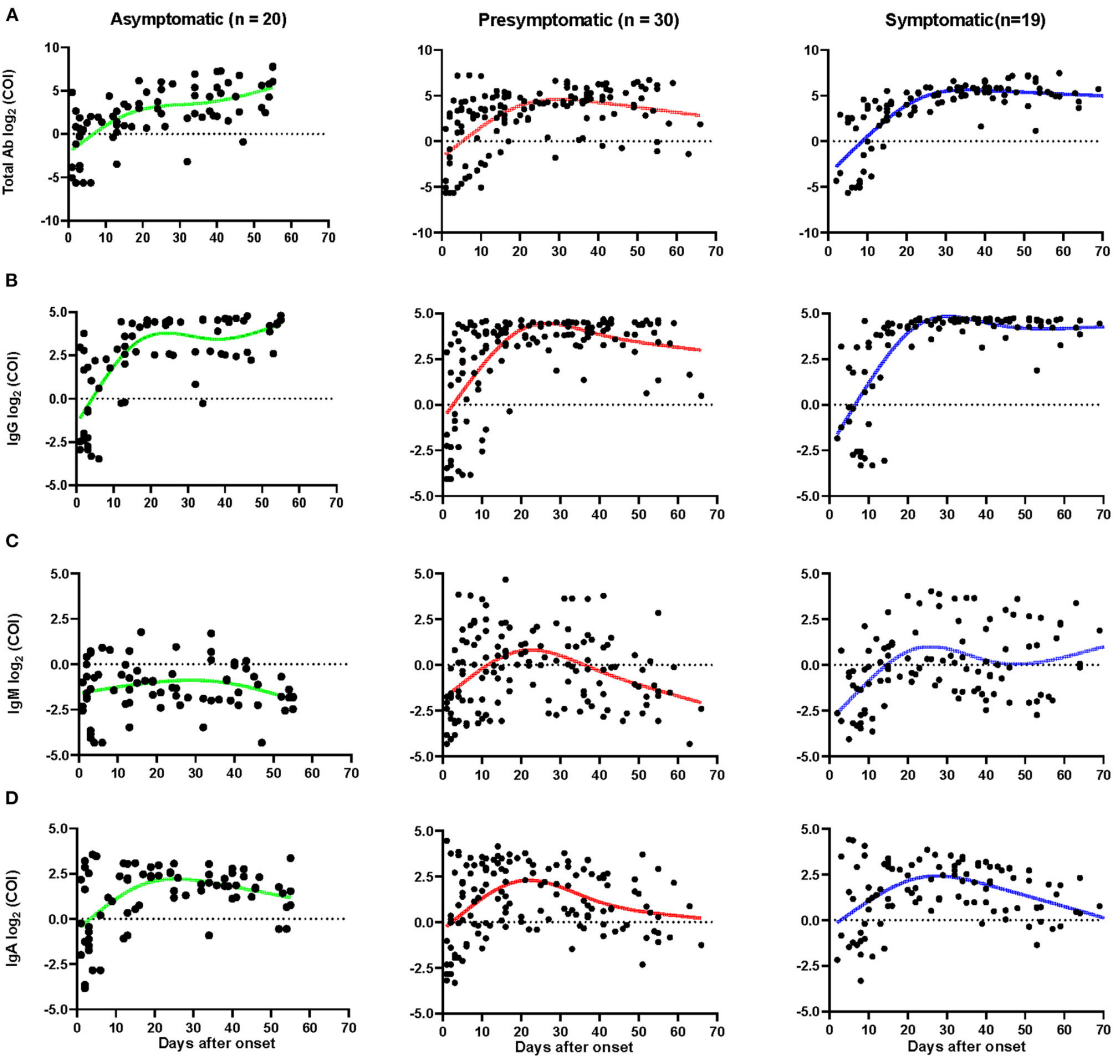
Dynamics of SARS-CoV-2 specific antibodies. The levels of total Ab **(A)**, IgG **(B)**, IgM **(C)**, and IgA **(D)** of different patients after onset. The relative antibody level was estimated using log_2_ (COI). Each dot represents a sample, curves represent the best fit line. Fit Spline/LOWESS of XY analyses with default ones was used for the fitted curve. Patients with totally negative antibodies or only one sample were excluded. Negative results are shown below the dotted horizontal lines.

A total of 324 plasma samples were detected for total Ab, IgM, IgG, and IgA against SARS-CoV-2, including samples from 77 asymptomatic carriers, 142 pre-symptomatic patients, and 105 symptomatic patients. The total seropositive conversion rate for Ab, IgG, and IgA of asymptomatic patients was 90.9% (20/22), 95.5% (21/22), and 90.9% (20/22), respectively (Supplementary Table 1); pre-symptomatic patients with 93.8% (30/32), 93.8% (30/32), and 90.6% (29/32); and all symptomatic patients experienced seropositive conversion of total Ab, IgG, and IgA. As shown in Figure 4, there was no obvious difference of total Ab, IgG, and IgA among the three groups (*p* > 0.05). Nearly half of cases had total Ab, IgG, and IgA within 1–7 days since the onset and more cases had antibodies within 8–14 days then almost all of the cases had these antibodies within 15–30 days and were maintained with time (Figures 3A,B,D). Notably, none of the asymptomatic carriers loss IgA, while 6 (21.4%) of pre-symptomatic and 3 (15.8%) symptomatic patients lost IgA during follow-up.

The total seropositive conversion rate of IgM reached 45.5% (10/22), 62.5% (20/32), and 63.2% (12/19) for asymptomatic, pre-symptomatic, and symptomatic patients (Supplementary Table 1), respectively. Importantly, IgM varied widely among the three groups. In pre-symptomatic and symptomatic patients, 30.4 and 12.5% of cases had IgM within the first week after onset of illness and the cases with IgM were increased until their peak 15–30 days after onset of illness (71.4 and 64.7%) and were then gradually decreased. The seropositive conversion rate of IgM decreased to 55 and 41.7% for pre-symptomatic and symptomatic patients, respectively, at 1–2 months after admission. By contrast, only 20% of asymptomatic cases produced IgM during the first week of admission, and most the cases failed to produced IgM in subsequent follow-up periods (Figure 3C).

### Dynamics of Antibody Response With Time

The IgG peaked as early as 20 days after the onset of admission for asymptomatic cases, but the pre-symptomatic and symptomatic patients peaked later (Figures 4A–D). The average number of days until peak IgG COI of asymptomatic cases was 3.5 days, which was lower than symptomatic patients, which peaked at COI of 4.5 (*p* < 0.05, Supplementary Table 2). The IgG was found to last in all three groups for 2 months or longer. Similar dynamics occurred for total Ab responses in the three groups (Figure 4A). Most of the asymptomatic cases had undetectable IgM, with concentrations varying slightly over time. Pre-symptomatic and symptomatic patients had higher IgM levels and some of them had persistent IgM levels for more than 70 days (Figure 4C). Compared with symptomatic patients, the peak IgM was significantly lower, with a COI of 0.84, in asymptomatic cases (*p* < 0.05, Supplementary Table 2). No obvious differences were observed in the dynamics of IgA among the three groups (Figure 4D), though the IgA peaked sooner in asymptomatic and pre-symptomatic patients, compared to symptomatic participants.

## Discussion

Although earlier studies aimed to understand the infectiousness of asymptomatic carriers (8, 10, 11), the virological and immunological dynamics in these patients remain elusive. In this study, the clinical and laboratory features of asymptomatic, pre-symptomatic, and symptomatic SARS-CoV-2 infected patients were quantitatively described and analyzed.

As highlighted in recent studies, COVID-19-specific mortality is age-related, with deaths mainly occurring in patients over 60 years old; while young patients usually present with moderate or mild manifestations of the disease (12). The same was reported in this study, as the median age of asymptomatic patients was 30, and half of them were children. One possible reason for asymptomatic COVID-19 cases to be more common in young adults is that a child or young person has a number of naïve immune cells, which can be easily educated by new antigens. By contrast, older populations have a limited number of naïve immune cells, thus making them more susceptible to severe COVID-19 disease (13). Since that young people are more likely to be asymptomatic, thus they must be targeted in preventative efforts to assure proper precautions are taken and reduce the potential for transmission, especially in the context of schools.

Notably, there were 13 asymptomatic patients with abnormal chest CT scans, which was common in other studies as well (14, 15). This suggests the possibility of lung damage even if an individual is asymptomatic and not experiencing any other symptoms. Or the lung damage was due to other co-morbidities. Others have suggested that a chest CT be the first choice in the screening of close contacts and patients (16), which may be helpful for the early diagnosis and treatment of infected patients. Especially as not all patients experience the signs and symptoms of COVID-19, using additional tools such as CT screenings may be more efficient and practical at capturing these high-risk transmitters.

For the viral dynamics of SARS-CoV-2, there were no obvious differences in the calculated initial Ct values among the three groups. Interestingly, as the fitted curve showed, the model-based initial viral load in nasopharyngeal swabs of asymptomatic carriers was the lowest, followed by symptomatic and pre-symptomatic patients. This may be related to the severity of the disease, with symptomatic patients progressing to more severe stages of disease (17, 18). In addition, the RNA negative-conversion of asymptomatic carriers occurred 15–20 days after admission, which occurred later for pre-symptomatic and symptomatic patients. Thus, it could be concluded that asymptomatic carriers show an earlier viral clearance. Additional noteworthy was that the viral RNA from two asymptomatic children was still detectable 50 days after admission, indicating continual shedding of the SARS-COV-2 virus. We also observed a re-appearance of SARS-CoV-2 virus RNA in eight asymptomatic patients after discharge, of whom had a positive RNA test up to 54 days after discharge, indicating they may continue shedding SARS-CoV-2 virus for a long time. Based on these observations, asymptomatic carriers showcased a large variation in viral dynamics. Therefore, vigilant control measures must be continued at this stage of the COVID-19 epidemic to avoid a resurgence caused by asymptomatic cases.

Asymptomatic, pre-symptomatic, and symptomatic patients all showed a rapid increase in IgG within 7 days of symptom onset, confirming previous report (19, 20). Importantly, most asymptomatic cases had constantly low levels of IgM, but high levels of IgG, and an earlier viral clearance. One possibility is the asymptomatic cases did not experience an acute phase, or they may have already experienced the acute phase before they were discovered by active surveillance. Furthermore, half of the asymptomatic carriers are child and the vast majority of children had low levels of IgM. Future studies should investigate the intricate relationship of IgM, age, and clinical outcomes in order to improve control strategies.

This study has several limitations, such as single-center retrospective study, a possible selection bias of enrolled patients due to only government-mandated COVID-19 facility, and undefined roles of low levels of IgM in asymptomatic carriers.

Taken together, we found that asymptomatic carriers were younger, with a lower initial viral load, early viral clearance, mild laboratory changes, mild chest CT manifestations, undetectable IgM, and moderate levels of IgG. This sheds light on the management and potential immune mechanisms of viral clearance in asymptomatic carriers, useful tools that are needed to improve and strengthen existing care guidelines.

## Methods

### Study Design and Participants

This was a retrospective analysis of patients with confirmed COVID-19 from the Third People's Hospital of Shenzhen between January 23, 2020, and April 1, 2020. Inclusion criteria were patients who tested positive for the SARS-CoV-2 virus or had serum antibodies to the virus. Cases were divided into asymptomatic, pre-symptomatic, or symptomatic cases based on the patients' symptoms and disease-related outcomes. The asymptomatic and pre-symptomatic patients enrolled in this study were identified through active surveillance and contact tracing. Asymptomatic carriers showed no self-perceived or clinically recognizable symptoms from admission until 14 days post-discharge. Pre-symptomatic patients had no symptoms at admission, but gradually showed symptoms such as fever, cough, chest discomfort, diarrhea, headache, and myalgia during hospitalization. Symptomatic patients showed symptoms such as fever, cough, chest discomfort, diarrhea, headache, and myalgia at admission. The discharge criteria for recovered patients were: (1) normal temperature for more than 3 days; (2) respiratory symptoms significantly improved, classified as significant absorption of pulmonary lesions on chest computerize tomography (CT) scans; (3) two consecutive negative ribonucleic acid (RNA) tests in 24 h. Abnormal chest CT was the presence of unilateral lobe lesions, multiple lobes in both lungs, or all lobes in both lungs. Finally, 23 asymptomatic, 33 pre-symptomatic, and 19 age-matched symptomatic patients were selected. This study was approved by the Ethics Committee of Shenzhen Third People's Hospital (2020-169) and all patients gave their oral consent to participate in this study.

### Data Collection

The medical records of 449 COVID-19 patients were reviewed. The epidemiological, demographic, clinical, and laboratory data of these patients were retrospectively collected. According to the Guidelines for the Diagnosis and Treatment for Novel Coronavirus Pneumonia (the Seventh Edition) published by the National Health Commission of the People's Republic of China (6), all diagnosed cases of COVID-19 were confirmed using respiratory reverse transcription polymerase chain reaction (RT-PCR) tests or antibody tests for SARS-CoV-2.

Ribonucleic acid (RNA) was extracted from 200 μL of respiratory or anal swab specimens using the Huayin-Bio Viral RNA Mini-Kit (Huayin, Shenzhen, China). The anal swab was perianal swabs of the skin about 2–3 cm from the anus. Samples for the SARS-CoV-2 virus were tested using the RT-PCR Kit (GeneoDX Co., Ltd., Shanghai, China) on an ABI 7500 thermocycler. Only the runs with valid internal references were included. Each RT-PCR assay provided a threshold cycle (Ct) value, which is the number of cycles required for the fluorescent signal to cross the threshold for a positive test, with a higher Ct value correlated to a lower viral load. The specimens were considered positive if the Ct value was <40.0, and negative if the viral load was undetectable. Specimen testing was repeated if the cycle-threshold value was higher than 37. The specimen was then considered positive if the repeat results were the same as the initial result and between 37 and 40. If the repeat Ct was undetectable, the specimen was considered negative. All procedures involving clinical specimens and SARS-CoV-2 were performed in a Biosafety Level 3 Laboratory.

The SARS-CoV-2 specific total antibody (Ab), IgG, IgA, and IgM in plasma was tested using a Chemiluminescence Microparticle Immuno Assay (CMIA). Briefly, antigens containing the receptor-binding domain (RBD) were used as the immobilized and HRP-conjugated antigen to detect total antibodies by double-antigens sandwich enzyme-linked immunosorbent assay (Ab-ELISA). IgM was tested by the IgM μ-chain capture method (IgM-ELISA), using the same RBD antigen as the Ab-ELISA. IgA and IgG were tested by the indirect ELISA using RBD antigen. The testing kits were supplied by the Beijing Wantai Biological Pharmacy Enterprise Co., Ltd., China. Fluorescence intensity was used to measure antibody concentration. The relative fluorescence of the sample to control (COI) was used to estimate the result. When COI was more than one, the result was judged to be positive.

### Statistical Analysis

The log2 transformation was done for COI. Variables were described using median, interquartile range (IQR), mean ± standard deviation (SD), or percentages. Chi-square or Fisher's exact tests were utilized to compare the proportions of the categorical variables. One-way ANOVA or Wilcoxon rank-sum tests were used for the continuous variables. Fit Spline/LOWESS of XY analyses with default ones was used for the fitted curve. The statistical tests were two-sided, and significant differences were considered at *P* < 0.05. All statistical analysis was performed using GraphPad Prism 8.3.0 (GraphPad Software, La Jolla, California, USA, www.graphpad.com).

## Data Availability Statement

The original contributions presented in the study are included in the article/Supplementary Material, further inquiries can be directed to the corresponding author/s.

## Ethics Statement

The studies involving human participants were reviewed and approved by Shenzhen Third People's Hospital. Written informed consent from the participants' legal guardian/next of kin was not required to participate in this study in accordance with the national legislation and the institutional requirements.

## Author Contributions

ZZ, LL, and ZW had the idea and for designed the study. ZZ, LL, TX, YW, and JY had full access to all data in the study and take responsibility for the integrity of the data and the accuracy of the data analysis. TX, YW, JY, SQ, HW, XL, ZW, LL, and ZZ had roles in the clinical management, patient recruitment, sample preparation, and clinical data collection. TX, LW, and HW had roles in the RNA and antibody detection experiments, data collection and analysis. TX, HY, and ZZ had roles in statistical analysis. TX, YW, LL, ZW, and ZZ had roles in data interpretation. TX and ZZ wrote the manuscript. LL, ZW, and ZZ contributed to critical revision of the report. All authors reviewed and approved the final version of the manuscript.

## Conflict of Interest

The authors declare that the research was conducted in the absence of any commercial or financial relationships that could be construed as a potential conflict of interest.

## Supplementary Material

Supplementary Figure 1 Dynamics of WBC, lymphocyte, CD4+ T, and CD8+ T cell counts. The total number of white blood cells (WBC), lymphocytes (LYMPH), CD4+ T, and CD8+ T cell tests at admission (Before) to hospital, after treatment (Middle), and before discharge (After) was shown. The red and purple dots represent two infants enrolled in this study.

Supplementary Table 1 Seroconversion rate of different antibodies in different patients.

Supplementary Table 2 Peak concentration and time of different antibodies.
