# Supplementary figures and images for: Early Viral Clearance and Antibody Kinetics of COVID-19 Among Asymptomatic Carriers
Source: Front Med (Lausanne). 2021 Mar 15;8:595773. doi: 10.3389/fmed.2021.595773 (PMC8005564; doi:10.3389/fmed.2021.595773)

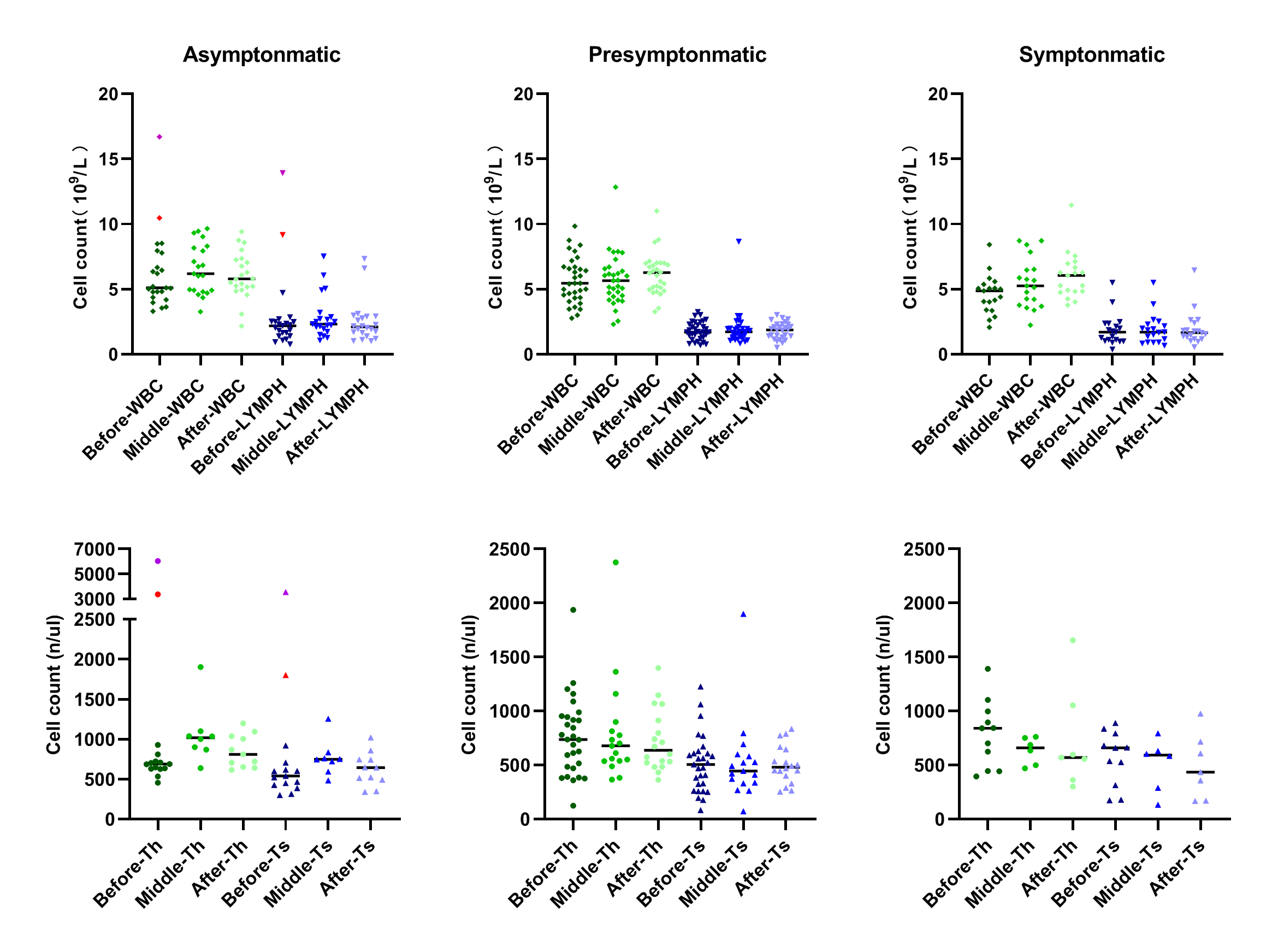

Supplement: Supplementary Figure 1 — Dynamics of WBC, lymphocyte, CD4+ T, and CD8+ T cell counts. The total number of white blood cells (WBC), lymphocytes (LYMPH), CD4+ T, and CD8+ T cell tests at admission (Before) to hospital, after treatment (Middle), and before discharge (After) was shown. The red and purple dots represent two infants enrolled in this study. [file Image_1.TIF]
